# Lower Critical Solution Temperature Phase Behavior and Water Activity of a Ternary Mixture of Oleic Acid, Lidocaine, and Water

**DOI:** 10.1021/acs.jced.5c00378

**Published:** 2025-10-07

**Authors:** Jordan D. Kocher, Ahmed Mahfouz, Hunter T. Bell, Joshua M. Rinehart, Akanksha K. Menon

**Affiliations:** † G.W.W. School of Mechanical Engineering, 1372 Georgia Institute of Technology, Atlanta, Georgia 30332, United States; ‡ School of Chemistry and Biochemistry, Georgia Institute of Technology, Atlanta, Georgia 30332, United States; § School of Materials Science and Engineering, Georgia Institute of Technology, Atlanta, Georgia 30332, United States

## Abstract

Mixtures that possess a lower critical solution temperature (LCST) phase behavior form a homogeneous single phase at temperatures below the LCST and separate into two liquid phases (water-rich, WR, and water-scarce, WS) above the LCST. This unique thermoresponsive phase behavior can be leveraged in various thermodynamic cycles, which are used for applications such as desalination and dehumidification. In addition to their phase diagram, the performance of aqueous LCST mixtures is dictated by their water activity (*i.e*., the chemical potential of water in the mixture). Recently, a ternary mixture of oleic acid (OA), lidocaine (LD), and water was shown to possess an LCST of ∼298.15 K, but the phase diagram over the full range of concentrations and water activity has not been reported. In this work, we experimentally characterize the phase diagram (liquid–liquid equilibrium, LLE), water activity (vapor–liquid equilibrium, VLE), chemical potential of water, and osmotic pressure of OA/LD/H_2_O under conditions that are relevant to the aforementioned applications. Our results suggest that OA/LD/H_2_O can outperform other LCST mixtures (*e.g*., ionic liquids) given its broad phase diagram, low LCST, and purity of the two phases after separation.

## Graphical abstract

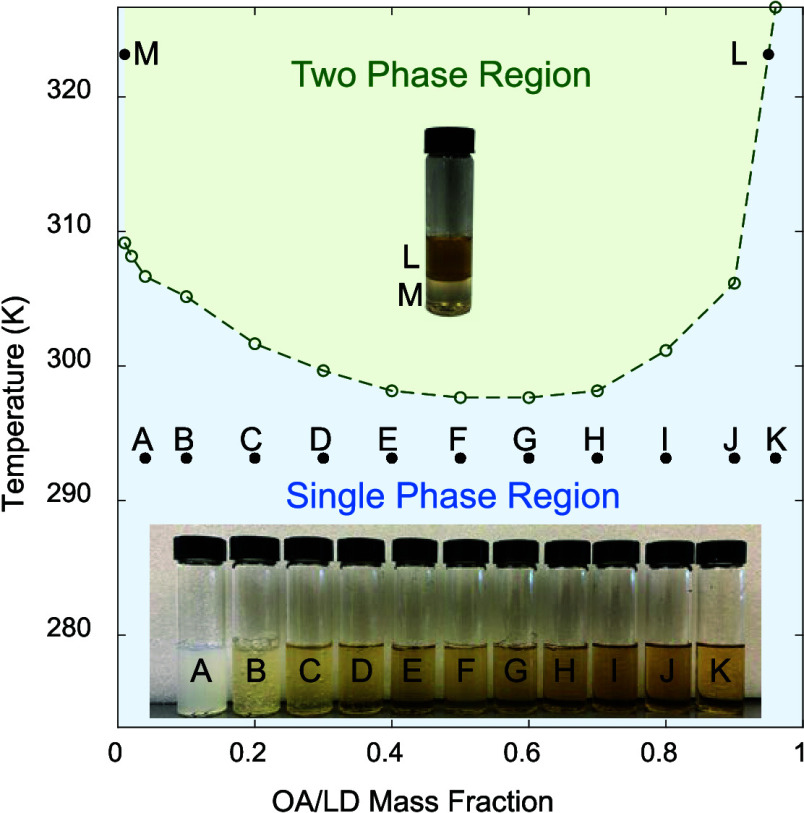

## Introduction

Mixtures that exhibit lower critical solution temperature (LCST) phase behavior are of interest for a range of emerging applications.^1–16^ LCST materials are typically characterized by a negative enthalpy and entropy of mixing due to the formation of ordered structures in solution.^17,18^ Aqueous LCST materials comprise temperature-dependent hydrophobic and hydrophilic moieties, resulting in separation into water-rich (WR) and water-scarce (WS) phases upon heating above the critical temperature. LCST hydrogels have been used in biomedical applications,^15,19–23^ while recent work has explored their use as desiccants for dehumidification and atmospheric water harvesting.^9,14,16,24–26^ Meanwhile, liquid mixtures that possess LCST (*e.g*., ionic liquids,^1,7,8,11,27^ polyelectrolytes,^2,28^ and deep eutectic solvents^10,29^ ) have been used for desalination,^12,13,30^ air conditioning,^10,31^ and dye separation.^29^

The general operation of a thermodynamic cycle utilizing aqueous LCST mixtures is illustrated in Figure 1. First, the single-phase homogeneous mixture is heated above a critical temperature to induce liquid–liquid phase separation. Then, the two phases (WR and WS) are physically separated and cooled to ambient temperature. The two separated phases are used to produce the desired effect (*e.g*., desalination, dehumidification, etc.), which brings them back into equilibrium. Finally, the two phases are recombined to complete the cycle. In the dehumidification cycle, the WR phase evaporates water to outdoor air (decreasing the concentration of water in the WR phase), while the WS phase absorbs moisture from indoor air (increasing the concentration of water in the WS phase).^10,32^ In the desalination cycle, LCST mixtures can be used as draw solutes for forward osmosis (FO)—freshwater spontaneously diffuses from a saline solution to the LCST mixture across a water-permeable membrane, after which heat is used to separate the diluted LCST mixture into a WS phase (regenerated draw solution) and a WR phase (nearly pure water).^12,33^ In these applications where an LCST mixture is used as the working fluid undergoing a thermodynamic cycle, only moisture (in the case of dehumidification) or liquid water (in the case of FO desalination) is introduced to the mixture, ensuring that the LCST material retains its phase behavior in the absence of any impurities or other ions.^10–12,32,34^

**Figure 1:**
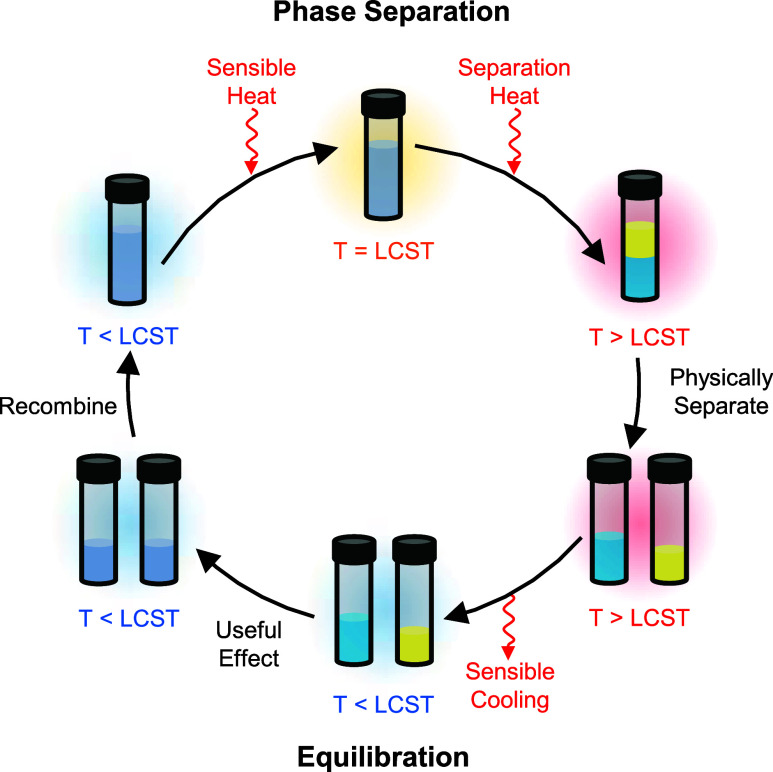
Illustration of a thermodynamic cycle using an LCST mixture. Phase separation first occurs upon heating above the LCST. Then, the mixture is physically separated at this higher temperature, followed by sensible cooling to ambient temperature. Finally, the two phases are used to produce a useful effect (*e.g*., desalination, dehumidification, etc.), which brings both phases to equilibrium.

In these thermodynamic cycles, the water activity (or chemical potential of water) of an LCST mixture determines the cycle performance. For dehumidification, the water activity of the WS phase determines the minimum indoor relative humidity that can be reached. Specifically, the WS activity at 298.15 K should be lower than 0.40 for dehumidification (to maintain an indoor relative humidity of 40% for thermal comfort).^35,36^ For FO desalination, the water activity determines the maximum salinity from which an LCST mixture can draw water spontaneously.^36^ As such, the WS activity should be lower than 0.75 for brine desalination (to draw water from a ∼25 wt % saline solution),^37^ and lower than 0.97 for seawater desalination (to draw water from a 3.5 wt % saline solution).^38^ However, many of the reported LCST mixtures separate into WS phases with relatively high water activities. For example, Kamio et al. explored a variety of aqueous LCST ionic liquids (binary mixtures of IL-water) with phosphonium and ammonium cations, and the lowest water activity was ∼0.95 in the WS phase of tetrabutylammonium trimethylbenzenesulfonate (N_4444_TMBS).^11^ Furthermore, for desalination, where clean water is the target output, the WR phase should have an activity approaching that of pure water (water activity of 1). For these LCST ILs reported in the literature, an additional purification step (*e.g*., nanofiltration) is required to remove residual ionic liquid from the WR phase. There is thus a need for new LCST mixtures that yield WS phases with low water activities and WR phases with near unity activities.^36^

Longeras et al. discovered that a deep eutectic solvent (DES) consisting of oleic acid (OA), lidocaine (LD), and water exhibits LCST, which they attribute to a significant change in ionic state with temperature.^29^ The thermoresponsive behavior of this ternary mixture (OA/LD/H_2_O) was used for dye separation. In that work, the phase separation temperature of ternary mixtures at an OA:LD mass ratio of 1:1 was reported at four different concentrations in water (10, 25, 75, and 90 wt %).^39^ Thereafter, Meyer et al. reported the full phase diagram of this DES for a biocatalytic application.^40^ However, the water activities of these mixtures have not been characterized, which is important to assess their performance for other applications, including desalination, atmospheric water harvesting, refrigeration, and dehumidification. Recently, we used the OA/LD/H_2_O mixture for dehumidification and found that the WS phase had a water activity of ∼0.87,^10^ which is significantly lower than the LCST ionic liquids reported in the literature. This promising result motivates the characterization of its thermodynamic properties with the goal of leveraging its thermoresponsive phase behavior for energy and water applications.

In this work, we report the phase diagram of OA/LD/H_2_O (liquid–liquid equilibrium or LLE), as well as the water activity (vapor–liquid equilibrium or VLE), chemical potential, and osmotic pressure of the ternary mixture at different concentrations in water (OA:LD mass ratio of 1:1). This data can be used to model the performance of various thermodynamic cycles based on OA/LD/H_2_O as the working fluid. Compared to other LCST mixtures reported in the literature, we find that the phase diagram of OA/LD/H_2_O is much broader, which leads to very pure WR and WS phases (water activity of 0.997 in the WR and 0.894 in the WS) that enable its use in energy-water applications.^36^

## Experimental Methods

Lidocaine was purchased from the Tokyo Chemical Industry (L0156-500G). We first purchased 25 mL of oleic acid from Tokyo Chemical Industry (O0180-25 ML), and then purchased a larger volume from Lab Alley (C5820-500 mL). For all of the aqueous mixtures, deionized water was used. We prepared one OA/LD/H_2_O mixture with the oleic acid from TCI and another with the oleic acid from Lab Alley. We found that the water activities of the two mixtures were comparable, confirming that the significantly less expensive Lab Alley oleic acid yielded the same performance. All of the data reported in this work correspond to mixtures prepared with the Lab Alley oleic acid. Table 1 lists the chemicals used along with their purities, suppliers, and CAS numbers.

**Table 1:** Chemical Components, CAS Registry Numbers, Suppliers, and Component Purity of All Chemicals Used in This Work

component	CAS reg. no.	suppliers	supplier ID no.	mass purity
oleic acid	112-80-1	Lab Alley	C5820-500 mL	>95%
lidocaine	137-58-6	Tokyo Chemical Industry	L0156-500G	>99.0%

When preparing mixtures with very low or very high wt % of OA/LD in water (*i.e*., 1, 2, 4, 96, 98, and 99 wt % OA/LD), we used a high-resolution analytical balance (MS204S scale from Mettler Toledo). After the samples were prepared, the vials were placed in a water bath set to 343.15 K for 24 h to ensure that all of the lidocaine dissolved and the samples were fully equilibrated. Then, the samples were removed from the water bath and cooled to room temperature (∼293.15 K). Since all of the samples phase separate at 343.15 K (except for the samples with 1, 2, 98, and 99 wt % water), the vials were shaken after they cooled back to room temperature and were then allowed to sit for at least 24 h, such that both phases were completely mixed and the samples reached equilibrium. Only after this procedure, the water activities and phase separation temperatures of the samples were measured.

To measure the water activity, we used the Aqualab 4TE water activity meter (Addium Inc.). All activity measurements in this work were taken at 293.15 and 298.15 K in “Custom” mode, where at least six measurements were taken until the value changed by less than 0.003 between the two most recent measurements. The activity meter automatically equilibrates the temperature of the sample to the set point before performing a measurement. However, a subsequent run in this Custom mode will yield another group of readings with a mean that may deviate from the mean of the previous run. As such, for a given sample, we performed multiple runs in Custom mode until the mean of the measurements from the latest run was within 0.003 of the mean of the measurements from the previous run. The water activity meter was calibrated using two different solutions of known water activity (LiCl/H_2_O and NaCl/H_2_O) prior to use.

To obtain liquid–liquid equilibrium (LLE) data for the WR and WS phases of the OA/LD/H_2_O mixture at different temperatures, the LCST phase diagram was characterized using temperature-controlled ultraviolet–visible spectroscopy (Agilent Technologies Cary 5000 UV–vis) at 600 nm, as it is a standard protocol for LCST materials.^41^ For an LCST material, clouding occurs as the phase separation temperature is approached, at which point the mixture transitions from a single homogeneous phase to the WR and WS phases.^41^ The sample is pipetted into a cuvette, which is placed within a temperature-controlled jacket. The temperature of the jacket is controlled via a Varian Cary Dual Cell Peltier Accessory. The sample temperature was initially set to 293.15 K. After holding the temperature constant for 120 s (equilibration time), it was increased by 0.5 K and then held constant again for 120 s. This procedure was repeated, increasing the sample temperature in 0.5 K increments, until the sample clouded, and a corresponding decrease in transmission was recorded. The temperature at which the transmission dropped below 5% was taken as the phase separation temperature for a given concentration. For certain concentrations, clouding was visually observed at the bottom of the vial (because of the nonuniform heating within the UV–vis), but it did not propagate to the top of the vial, where the transmission measurement is made. For these samples, the phase separation temperature was recorded as the temperature at which clouding was visually observed and not the temperature at which a drop in transmission was measured by the UV–vis. This procedure was then repeated for the entire range of concentrations that were prepared; the locus of these points then forms the binodal curve in the phase diagram. Samples containing 1, 2, 98, and 99 wt % water did not phase separate in the range of 293.15 to 373.15 K. As such, it was concluded that the binodal curve does not span these concentrations. The transmission vs temperature plots are shown in Supporting Note 1.

To quantify the viscosity of the LCST mixtures, a viscometer (RheoSense m-VROC) was used. A 2.5 mL sample of a given mixture was loaded into an m-VROC viscometer. The instrument was set to 298.15 K with a hold time of 3 min for temperature equilibration. The viscosity was then measured at a constant shear rate of 500 s^–1^ for 2 min. The reported temperature accuracy of the m-VROC viscometer is ±1 K with a viscosity accuracy of ±2%.

## Results and Discussion

The phase diagram of OA/LD/H_2_O ternary mixtures, representing the LLE between the WR and WS phases, is plotted in Figure 2a, showing an LCST of 297.6 ± 0.5 K at an OA:LD mass ratio of 1:1 across all concentrations in water (confirmed with NMR in Supporting Note 2). This is in close agreement with the phase data reported by Longeras et al., Meyer et al., and Corzo et al. at ∼298.15 K.^29,39,40,42^ The phase diagram is very broad, indicating that the two phases that the mixture separates into upon heating are very pure (*i.e*., the WR phase is nearly pure water and the WS phase is nearly pure OA/LD as dictated by the tie-rule and confirmed with NMR in Supporting Note 2).^30^ Notably, this phase diagram is considerably wider (1–96 wt % OA/LD) than other LCST mixtures reported in the literature;^11,12,27^ for example, the binodal curve of tetrabutylphosphonium trifluoroacetate in water (P_4444_TFA/H_2_O) only spans concentrations of 10–70 wt % P_4444_TFA.^12^ We note that the phase diagram shown in Figure 2a does not span ultrahigh concentrations since samples that had 98 and 99 wt % OA/LD did not cloud at temperatures up to 363.15 K.

**Figure 2:**
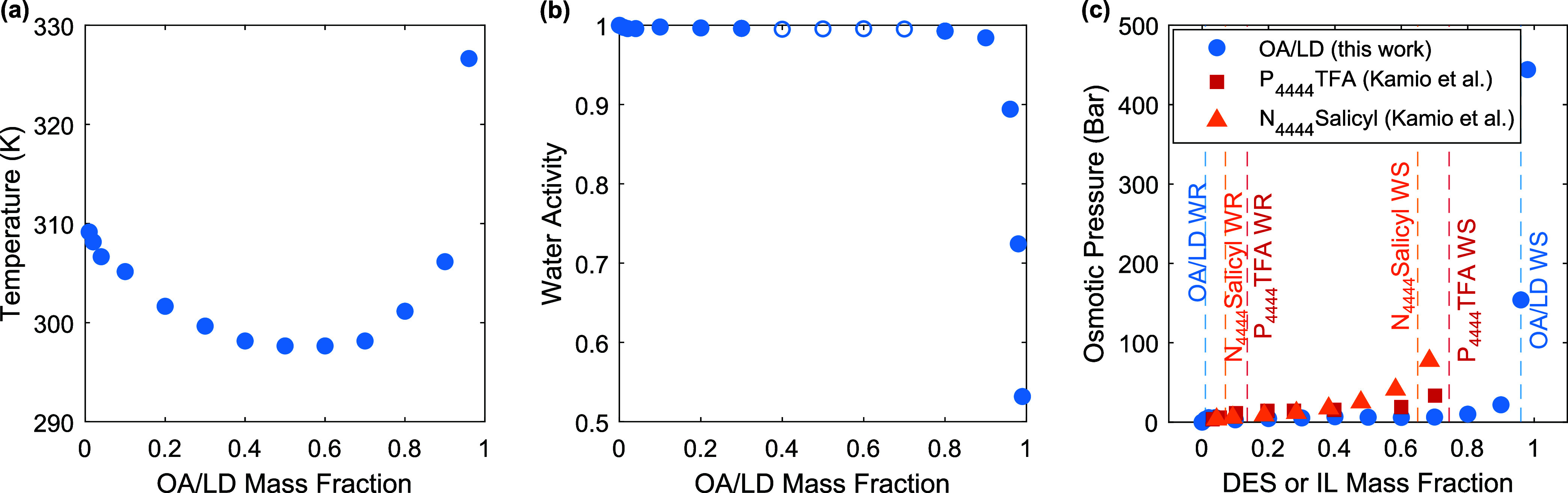
(a) Phase diagram of OA/LD/H_2_O as a function of the OA/LD mass fraction (mass fraction of OA + mass fraction of LD). The maximum error in the separation temperature measurements is 0.5 K. (b) Water activity of OA/LD/H_2_O, at 298.15 K, as a function of the OA/LD mass fraction. The mass ratio of OA:LD is maintained at approximately 1:1 across all concentrations in water for both the phase diagram and the activity measurements. (c) Osmotic pressure of aqueous mixtures of OA/LD, P_4444_TFA, and N_4444_Salicyl at 298.15 K. OA/LD is a deep eutectic solvent (DES), while the other two materials are ionic liquids (ILs). The dashed lines indicate the mass fractions of the WR and WS phases. The osmotic pressure of OA/LD is calculated from the activity in (b), while the osmotic pressure of the ILs is taken from Kamio et al.^11^ In (b) and (c), the water activity and osmotic pressure data are not reported at OA/LD mass fractions between 0.4 and 0.7 since the ternary mixture is biphasic at 298.15 K.

The water activity of OA/LD/H_2_O ternary mixtures is plotted in Figure 2b. Similar to IL/H_2_O binary mixtures that possess LCST,^11^ the water activity of the OA/LD/H_2_O ternary mixture is nearly constant for a large range of concentrations (1–70 wt % OA/LD). The activity decreases slightly from 70–90 wt % OA/LD, and only when the concentration exceeds 90 wt % OA/LD does the water activity begin to decrease significantly. We note that because these activity measurements were at 298.15 K, the samples at 40, 50, 60, and 70 wt % OA/LD were biphasic based on the phase diagram. In other words, the activity meter was measuring the activity of the two phases on the binodal curve at 298.15 K at these concentrations, and as such, these measurements were omitted as in Figure 2b. Measurements were also performed at 293.15 K, but we only present the data for 298.15 K in Figure 2 to allow for direct comparison with the osmotic pressure measurements of LCST ILs reported in the literature,^11,12^ which were at 298.15 K. The water activity measurements for all concentrations at both 293.15 and 298.15 K, as well as the corresponding chemical potential and osmotic pressure data, are provided in Table 2. Water activity values, *a*_*w*_, are expressed in equivalent chemical potentials of water, μ_w_, and osmotic pressures, Π, using eqs 1 and 2, respectively, where *R* is the universal gas constant, *T* is the temperature at which the activity was measured, and *v*_*w*_ is the molar volume of water.

1 $$\mu_{w}=\mathrm{RT}\;\ln\left(a_{w}\right)$$

2 $$\Pi =-\frac{\mathrm{RT}}{v_{w}}\ln\left(a_{w}\right)$$

**Table 2:** Phase Separation Temperature (*T*_b_), Water Activity (*a*_w_), Chemical Potential of Water (μ_w_), and Osmotic Pressure (Π) of OA/LD/H_2_O Mixtures at Different Mass Fractions of Water (*w*_w_), Oleic Acid (*w*_OA_), and Lidocaine (*w*_LD_) (OA/LD Mass Is Approximately Maintained at a 1:1 Ratio)^a^

				293.15 K			298.15 K		
*w*_w_	*w*_OA_	*w*_LD_	*T*_b_ (K)	*a*_w_	μ_w_ (J mol^–1^)	Π (bar)	*a*_w_	μ_w_ (J mol^–1^)	Π (bar)
0.010	0.495	0.495		0.5307	–1544	858.0	0.5318	–1565	869.6
0.021	0.490	0.489		0.7187	–805.0	447.2	0.7244	–799.3	444.1
0.040	0.480	0.480	326.65	0.8827	–304.1	168.9	0.8942	–277.2	154.0
0.099	0.450	0.450	306.15	0.9746	–62.61	34.78	0.9842	–39.38	21.88
0.201	0.400	0.400	301.15	0.9884	–28.44	15.80	0.9927	–18.11	10.06
0.300	0.349	0.350	298.15	0.9936	–15.75	8.749	0. 9953	–11.58	6.433
0.401	0.300	0.299	297.65	0.9946	–13.15	7.305	N/A	N/A	N/A
0.500	0.250	0.250	297.65	0.9958	–10.31	5.726	N/A	N/A	N/A
0.601	0.199	0.200	298.15	0.9958	–10.31	5.726	N/A	N/A	N/A
0.700	0.151	0.150	299.65	0.9956	–10.70	5.944	N/A	N/A	N/A
0.800	0.100	0.100	301.65	0.9954	–11.19	6.216	0.9967	–8.194	4.552
0.900	0.050	0.050	305.15	0.9968	–7.714	4.286	0.9979	–5.261	2.923
0.959	0.021	0.020	306.65	0.9967	–8.057	4.476	0.9959	–10.28	5.713
0.980	0.010	0.010	308.15	0.9967	–8.106	4.503	0.9959	–10.23	5.686
0.990	0.005	0.005	309.15	0.9990	–2.390	1.328	0.9974	–6.404	3.558

a N/A refers to concentrations that separated into two phases in the activity meter at 298.15 K.

Given the interest in utilizing LCST mixtures for forward osmosis desalination,^12,13^ we plot the osmotic pressure of OA/LD/H_2_O in Figure 2c as a function of DES mass fraction. For comparison, we also plot the osmotic pressures of ionic liquid LCST mixtures reported in the literature as a function of the IL mass fraction. For each mixture, the dashed lines denote the mass fraction of the two phases (WR and WS) after thermal separation at 343.15 K. We note that for N_4444_Salicyl, which has critical temperatures exceeding 343.15 K on the phase diagram, we interpolated to find the mass fractions associated with the WR and WS phases at this temperature. For OA/LD and P_4444_TFA, the phase diagram data ends at temperatures lower than 343.15 K, so we assume that the binodal curve of each mixture extends vertically from those end points. For example, in the data from Kamio et al., the highest temperature on the left side of the binodal curve of P_4444_TFA/H_2_O is 338.29 K, which corresponds to 13.6 wt % P_4444_TFA.^11^ As such, we assume that at any temperature greater than 338.29 K, the WR phase of P_4444_TFA/H_2_O consists of 13.6 wt % P_4444_TFA, and this value is used for the dashed line in Figure 2c.

From Figure 2c, it may initially appear that OA/LD/H_2_O would be a worse draw solution than the IL-based LCST mixtures because, at a given concentration, it has a lower osmotic pressure than the ILs (and a lower osmotic pressure indicates that it cannot draw water from high salinity feeds). However, upon observation of the dashed lines that indicate the WR and WS concentrations, it is clear that OA/LD/H_2_O separates into much purer WR and WS phases. This is particularly beneficial for desalination, in which a pure WR phase requires little post-treatment and a pure WS phase can draw water from higher salinity feeds. Notably, the WS phase of OA/LD/H_2_O has a higher osmotic pressure than that of any of the IL-based mixtures (2.4 × higher than N_4444_Salicyl). The higher osmotic pressure (*i.e*., lower water activity) of the WS phase also means that it can dehumidify air to lower humidities. Furthermore, the WR phase of OA/LD is >99 wt % water, whereas the WR phase of N_4444_Salicyl is only 93 wt % water, such that the WR phase of OA/LD would require less post-treatment to produce pure water. However, the high concentration in the WS phase of OA/LD will likely lead to other trade-offs, such as an increase in viscosity (which is undesirable from a system standpoint). To quantify this, the viscosities of the WS phases of OA/LD and N_4444_Salicyl were measured. The WS phase viscosities were 171.15 ± 7.94 mPa s for OA/LD and 26.50 ± 0.64 mPa s for N_4444_Salicyl at 298.15 K.

To confirm our phase diagram and water activity measurements, we prepared a mixture containing 25 wt % OA, 25 wt % LD, and 50 wt % H_2_O (*i.e*., an OA/LD mass fraction of 0.5 in water). At room temperature, the mixture was homogeneous as predicted by the phase diagram. Then, we heated the mixture in a water bath at 343.15 K for at least 12 h (after which the mixture is fully separated and the activity of the two phases does not change). After separating the WR and WS phases, we measured water activities of 0.9992 and 0.8903, respectively. We then dehydrated both samples in an environmental chamber at 323.15 K and 0% relative humidity for 12 h. By measuring the mass change after dehydration (with a 0.1 mg-scale resolution), we calculated that the WR phase was 99.0 ± 0.01 wt % H_2_O after separation, while the WS phase was 3.5 ± 0.01 wt % H_2_O. Given that this WS phase is phase separated at 343.15 K, the phase purity is slightly higher than the previously reported values by Longeras et al. at a temperature of 323.15 K (99.62 ± 0.1 wt % H_2_O and 5 ± 0.1 wt % H_2_O, respectively).^29^ These mass fractions are consistent with the phase diagram constructed using UV–vis cloud point measurements, while the activities correspond to our water activity measurements at those concentrations.

Since the LCST mixture characterized in this work is ternary, it is also important to determine if the ratio of OA/LD in the WR and WS phases is the same as the ratio of OA/LD in the initial single-phase mixture. Using a mass conservation analysis for the WS phase and NMR measurements for the WR phase, we confirmed that the ratio of OA/LD is approximately the same in both phases as it is in the single-phase mixture. This analysis is described in Supporting Note 2.

## Conclusions

Oleic acid, lidocaine, and water form a ternary mixture that possesses an LCST of 297.65 K when prepared with a 1:1 mass ratio of OA:LD. While this mixture has been previously used for dye separation^29^ and biocatalysis,^40^ water activity has not been reported, which has limited its use in other applications such as desalination and dehumidification. In this work, we characterize the phase diagram of this mixture (LLE), along with its water activity (VLE), chemical potential, and osmotic pressure. Notably, the OA/LD/H_2_O phase diagram is broader than other LCST materials (*i.e*., ionic liquids) reported in the literature, which results in very pure WR and WS phases upon separation. Furthermore, the activity of the WS phase of this ternary mixture is significantly lower than that of ionic liquid-based LCST mixtures, suggesting that OA/LD/H_2_O can yield improved performance in various thermodynamic cycles. Longeras et al. originally demonstrated that changing the OA:LD ratio alters the phase behavior of the mixture.^29^ Given this, and owing to its use in prior experimental demonstrations, we chose to investigate the 1:1 ratio to obtain the thermodynamic data needed across a large concentration range for quantifying system-level performance.^10,32^

## Supplementary Material



## Notes

Cloud point measurement procedure using UV–vis (Supporting Note 1), NMR procedure and spectra of all compounds (Supporting Note 2), and water activity measurement standard deviation (Supporting Note 3) (PDF)

## Abbreviations

IL: ionic liquid
LD: lidocaine
LCST: lower critical solution temperature
OA: oleic acid
WR: water-rich
WS: water-scarce
