# Supplementary material for: Lower Critical Solution Temperature Phase Behavior and Water Activity of a Ternary Mixture of Oleic Acid, Lidocaine, and Water
Source: J Chem Eng Data. 2025 Oct 7;70(11):4664–70. doi: 10.1021/acs.jced.5c00378 (PMC12621186; doi:10.1021/acs.jced.5c00378)
Supplement: Supplementary file 1 [file je5c00378_si_001.pdf]

Supplementary Information

Lower critical solution temperature phase behavior and water activity of a ternary mixture of oleic acid, lidocaine, and water

Jordan D. Kocher[1], Ahmed Mahfouz[1], Hunter T. Bell[2], Joshua M. Rinehart[3], and Akanksha K. Menon[1*]

[1]George W. Woodruff School of Mechanical Engineering, Georgia Institute of Technology, Atlanta, GA 30332, USA

[2]School of Chemistry and Biochemistry, Georgia Institute of Technology, Atlanta, GA 30332, USA

[3]School of Materials Science and Engineering, Georgia Institute of Technology, Atlanta, GA 30332 USA

*Corresponding author. Email: akanksha.menon@me.gatech.edu

Supplementary Note 1: Cloud Point Measurement Procedure

The transmission vs. temperature plots are shown in Fig. S1. At 4 wt.% water, the mixture did not completely cloud; however, a small "wisp" of clouding was observed (see Fig. S2) when the temperature was increased above a certain value. After maintaining the 4 wt.% water sample at this increased temperature for 12 hours, macroscopic phase separation was observed in the form of a small droplet of the water-rich phase at the bottom of the vial. This confirms that the slight visual clouding was due to phase separation. It is likely that there was too little water for the entire sample to cloud, resulting in only a small portion of visible clouding. We then cooled the 4 wt.% water sample down to ambient temperature, allowed it to re-mix, and placed it in a water bath. We increased the water bath temperature until the wisp of clouding was observed; the first temperature at which this wisp was observed was 53.5 °C, which we take to be the phase separation temperature for 4 wt.% water. The samples containing 1 and 2 wt.% water did not phase separate at all in the range of 20 – 90 °C. The locus of the points at which separation was observed forms the binodal curve in the phase diagram.

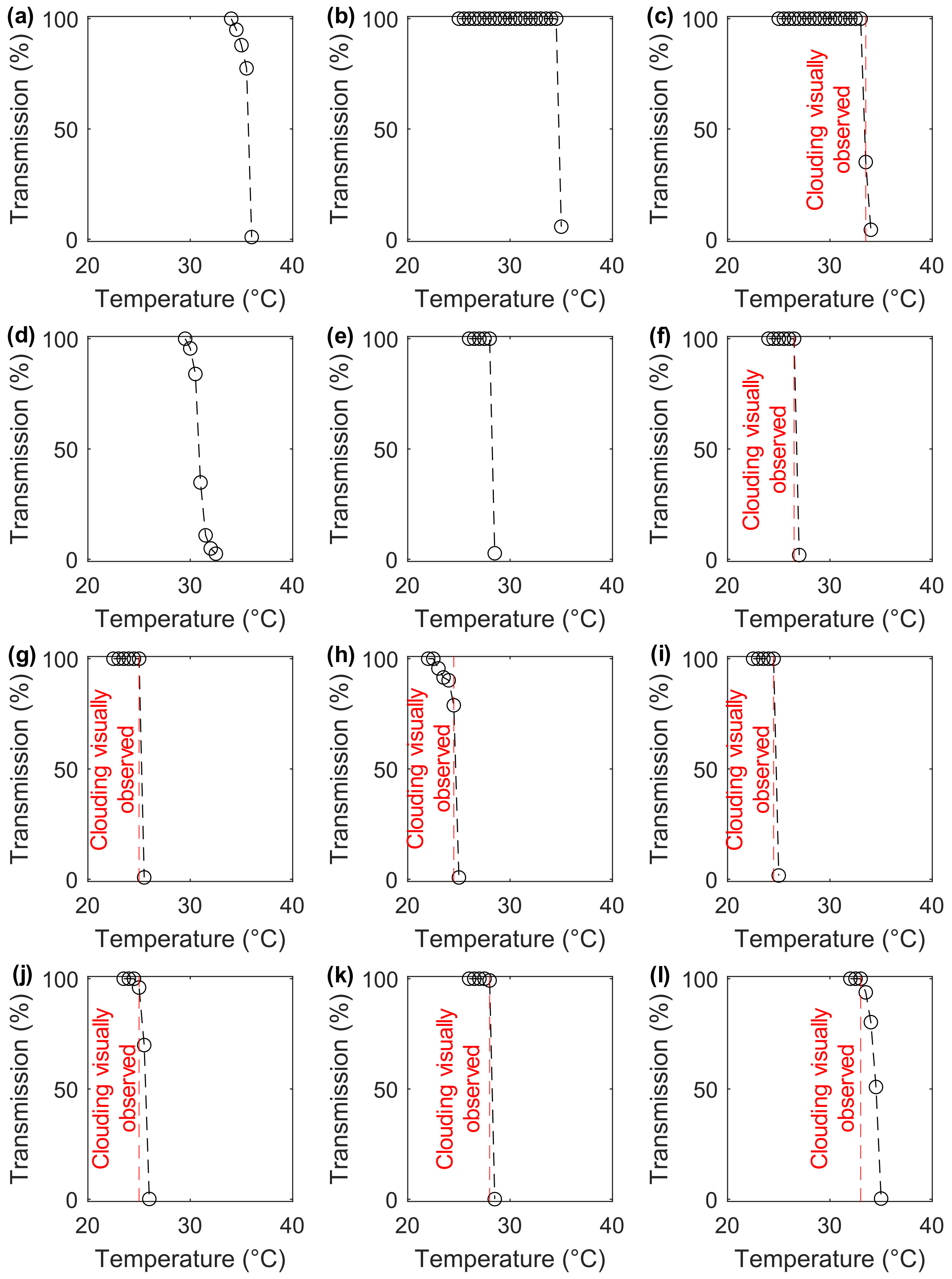

Fig. S1. Transmission vs temperature plots from UV-Vis. (a) 1 wt.% OA/LD; (b) 2 wt.% OA/LD; (c) 4 wt.% OA/LD; (d) 10 wt.% OA/LD; (e) 20 wt.% OA/LD; (f) 30 wt.% OA/LD; (g) 40 wt.%

OA/LD; (h) 50 wt.% OA/LD; (i) 60 wt.% OA/LD; (j) 70 wt.% OA/LD; (k) 80 wt.% OA/LD; (l) 90 wt.% OA/LD. Red dashed vertical lines correspond to the temperatures at which clouding was visually observed at the bottom of the vial but did not propagate high enough to reach the point where transmission was measured by the UV-Vis.

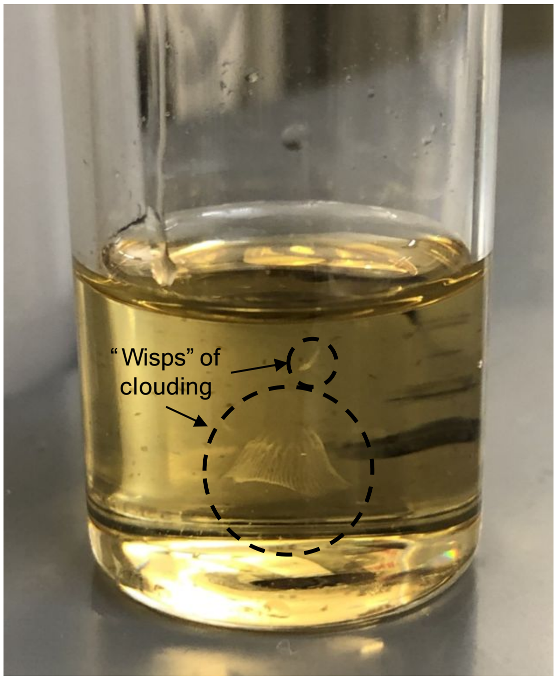

Fig. S2. Phase separation in OA/LD/H_2O with a very low concentration of water (4 wt.%).

Supplementary Note 2: NMR Procedure and Spectra for All Compounds

Since the OA/LD/H_2O mixture in this work is ternary, we investigated whether the ratio of OA to LD in the WR and WS phases (after separation) is the same as in the single-phase mixture (before separation). We obtained the phase diagram in Figure 2a of the main text using data only from mixtures prepared with equal parts by mass OA and LD. However, it is possible that a single-phase mixture (*e.g.*, one containing 25 wt.% OA, 25 wt.% LD, and 50 wt.% H_2O) could phase separate into a mixture containing a WR phase with more OA than LD and a WS phase with more LD than OA (or vice versa) when heated. To further understand this, we can use a simple mass balance on the WS phase. Because we observed that the WR phase has very little OA and LD (the WR phase is 99 wt.% H_2O), this means that nearly all of the OA and LD within the original, single-phase mixture will end up in the WS phase. In this case, the WS phase will possess approximately the same ratio of OA to LD as the original mixture (*i.e.*, if the mass ratio is 1:1 OA to LD in the initial mixture, the WS phase must also be nearly 1:1 OA to LD). However, this mass balance cannot be used to calculate the OA to LD ratio in the WR phase. Instead, we used NMR to measure the mole fraction of the different species within the WR, and we found that the OA to LD ratio is 1.08:1 (very nearly 1:1); see Fig. S4 below. These results suggest that an OA/LD/H_2O mixture initially prepared with a 1:1 mass ratio of OA to LD will separate into phases that remain at nearly 1:1 ratios of OA to LD, such that our results in Figure 2 of the main text can be used to find the properties of the WR and WS phases.

^{1}H NMR was performed with a Bruker Avance III (400 MHz) on the water-scarce (WS) and water-rich (WR) phases of OA/LD/H_2O. Initially, a 25/25/50 wt.% mixture of OA/LD/H_2O was heated for > 12 hrs., after which the WR and WS phases were physically separated. A few drops (≈ 10 mg) of each phase were placed in separate vials. Anhydrous deuterated acetonitrile

(ACN-d3) was utilized as the solvent for ^{1}H NMR, due to its complete miscibility with all mixture components. The ^{1}H NMR of the WS phase is shown in Fig. S3. Peaks are color coded to the compounds and appropriately labeled. Integrations are carried out such that the OA methyl group integrates to 3 protons. The rest of the OA peaks integrate very near to the expected value. The relative integration for LD can be then used to determine the mole fraction and weight fraction of the mixture. Note that the peak for water is found at 3.2 ppm and overlaps with 2 protons on LD. This peak integration can be broken up in the representative LD and H_2O integrations. Unnormalized mole fractions are calculated by dividing the integrated value of a peak by the true number of protons associated with that peak. For example, utilizing the integrations of 3 for OA (0.93 ppm), 3.73 for LD (7.11 ppm), and 0.12 for H_2O (3.20 ppm), the unnormalized mole fractions are calculated as $3x = 3$, $3y = 3.73$, $2z = 0.12$, where x, y, and z are unnormalized mole fractions for OA, LD, and H_2O, respectively. The peaks can be normalized to mole fractions by $\frac{x}{x+y+z} = X_{OA} = 0.435$, $\frac{y}{x+y+z} = X_{LD} = 0.539$, and $\frac{z}{x+y+z} = X_{H_2O} = 0.026$. Taking account of molecular weight yields weight fractions of $w_{OA} = 0.492$, $w_{LD} = 0.506$, and $w_{H_2O} = 0.002$. This yields an OA to LD mass ratio of 0.97:1 (nearly 1:1, as expected). Notably, the mass fraction of H_2O is less than expected for the WS phase (based on the results in the main text). However, this could have occurred due to water loss (via evaporation) from the WS mixture during separation or storage before the ^{1}H NMR took place.

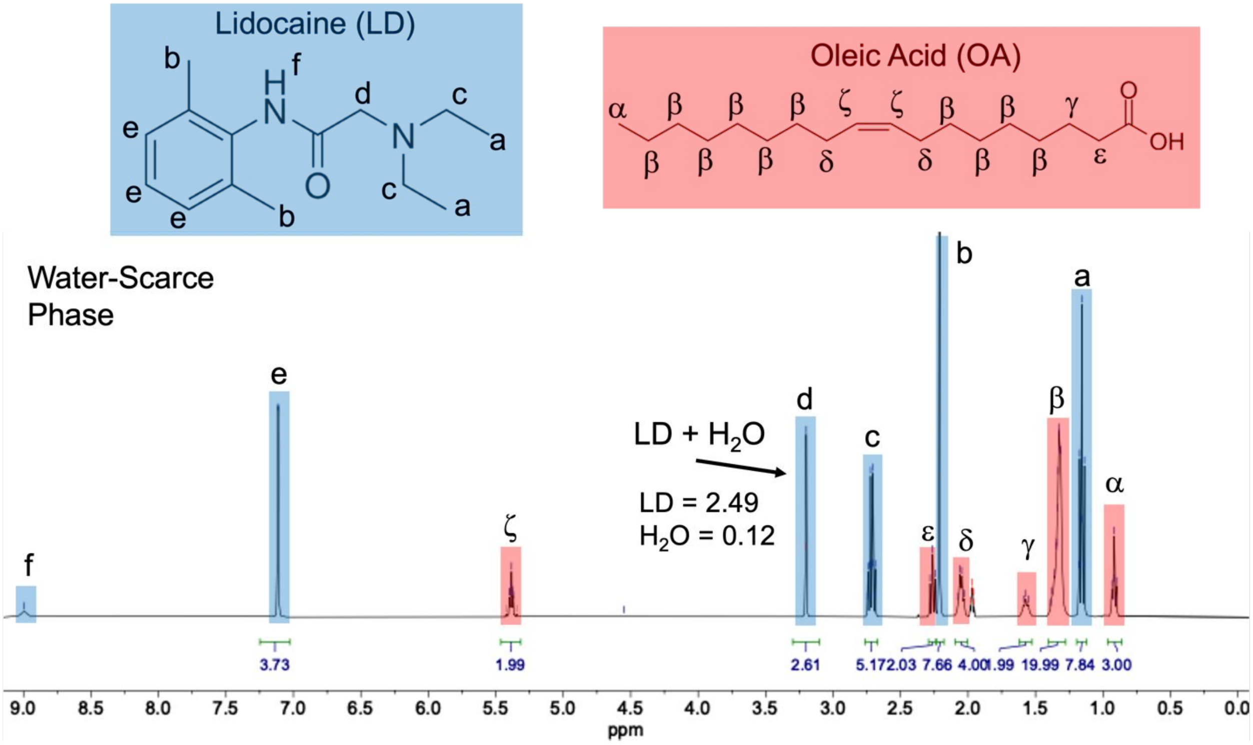

Fig. S3. ^{1}H NMR of water scarce phase, showing peak assignments and integrations.

In a similar way, the composition of the WR phase can be calculated according to integrations shown in Fig. S4. Peak assignments remain the same as Fig. S3. Normalization of the integrations into mole fractions leads to $X_{OA} = 8.45 \times 10^{-4}$, $X_{LD} = 9.47 \times 10^{-4}$, and $X_{H_2O} = 0.998$. This corresponds to weight fractions of $W_{OA} = 0.013$, $W_{LD} = 0.012$, and $W_{H_2O} = 0.975$. This yields an OA to LD mass fraction of 1.08:1.

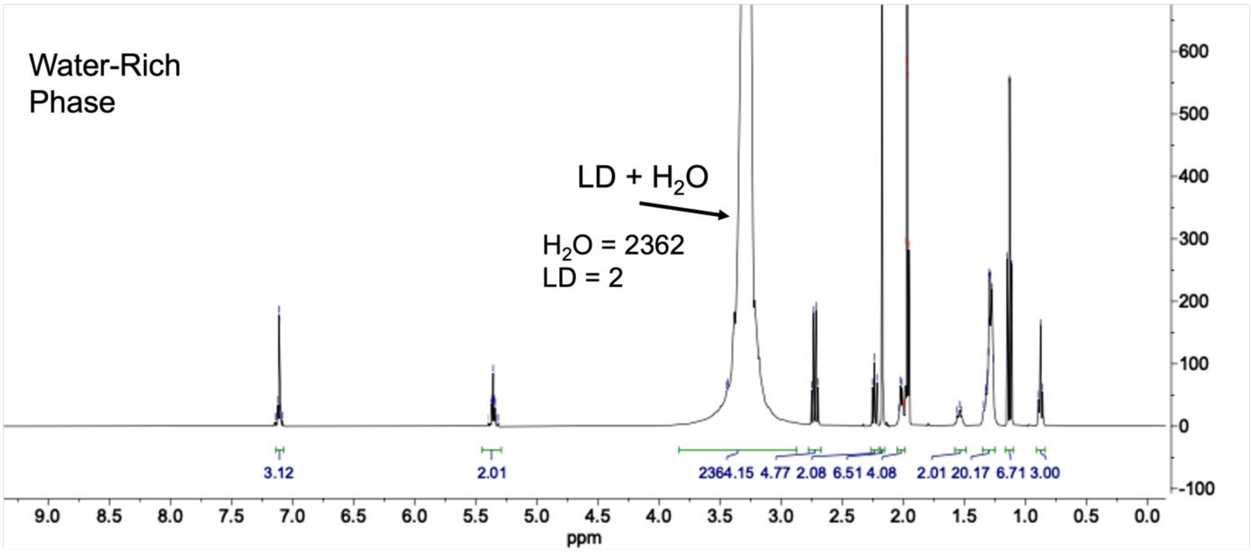

Fig. S4. ^{1}H NMR of water rich phase.

Supplementary Note 3: Water Activity Measurement Standard Deviations

Herein, we report the standard deviations associated with the activity measurements shown in Figure 2b and Table 2 of the main text. Five consecutive activity measurements were taken for each sample at a given temperature and composition. The values reported in Figure 2b and Table 2 of the main manuscript represent the mean of those five measurements. Table S1 reports the standard deviations of those five consecutive activity measurements. We note that the standard deviation across all activity measurements is 3-4 orders of magnitude smaller than the measured activity of each sample.

Table S1. Standard deviation and 95% confidence interval (CI) of OA/LD/H_2O activity measurements (a_w) reported in Figure 2b and Table 2 of the main manuscript at 20 °C and 25 °C. OA to LD mass is approximately maintained at a 1:1 ratio, and a_w is measured at different mass fractions of water. N/A refers to concentrations that phase separated in the activity meter at 25 °C.

			20 °C		25 °C	
w_w	w_{OA}	w_{LD}	a_w (Std. Dev.)	0.95 CI	a_w (Std. Dev.)	0.95 CI
0.010	0.495	0.495	0.00023	0.00026	0.00070	0.00079
0.021	0.490	0.489	0.00030	0.00034	0.00028	0.00032
0.040	0.480	0.480	0.00052	0.00059	0.00048	0.00054
0.099	0.450	0.450	0.00036	0.00041	0.00042	0.00047
0.201	0.400	0.400	0.00016	0.00018	0.00017	0.00019
0.300	0.349	0.350	0.00051	0.00057	0.00022	0.00025
0.401	0.300	0.299	0.00030	0.00034	N/A	N/A
0.500	0.250	0.250	0.00038	0.00043	N/A	N/A
0.601	0.199	0.200	0.00038	0.00043	N/A	N/A
0.700	0.151	0.150	0.00038	0.00043	N/A	N/A
0.800	0.100	0.100	0.00026	0.00029	0.00050	0.00056
0.900	0.050	0.050	0.00070	0.00079	0.00042	0.00047
0.959	0.021	0.020	0.00022	0.00025	0.00026	0.00029
0.980	0.010	0.010	0.00065	0.00073	0.00052	0.00059
0.990	0.005	0.005	0.00040	0.00045	0.00050	0.00056
